# Development of *Lactococcus lactis* Biosensors for Detection of Sulfur-Containing Amino Acids

**DOI:** 10.3389/fmicb.2020.01654

**Published:** 2020-07-15

**Authors:** Jhonatan A. Hernandez-Valdes, Maximillian M. Dalglish, Jos Hermans, Oscar P. Kuipers

**Affiliations:** ^1^Department of Molecular Genetics, Groningen Biomolecular Sciences and Biotechnology Institute, University of Groningen, Groningen, Netherlands; ^2^MRC Laboratory of Molecular Biology, Cambridge, United Kingdom; ^3^Analytical Biochemistry, Department of Pharmacy, University of Groningen, Groningen, Netherlands

**Keywords:** *Lactococcus lactis*, methionine, cysteine, biosensor, auxotrophy, fluorescence, transcriptional sensor

## Abstract

The sulfur-containing amino acids methionine and cysteine play an important role in food industry. These amino acids are used to confer a sulfur smell or meat-related aroma to food products. Besides their use as food additives, methionine and cysteine participate in flavor formation in dairy fermentations. For instance, the characteristic aroma of Cheddar cheeses is derived from methionine. Therefore, bacterial strains with the ability to overproduce and secrete these amino acids are relevant for the food industry. In addition, the quantification of these compounds in food matrices is a laborious task that involves sample preparation and specific analytical methods such as high-performance liquid chromatography. The ability of bacteria to naturally sense metabolites has successfully been exploited to develop biosensors. The presence of a specific metabolite is sensed by the biosensors, and it is subsequently translated into the expression of one or more reporter genes. In this study we aim to develop biosensors to detect methionine and cysteine, which are produced and secreted by wild-type *Lactococcus lactis* strains. We employed two strategies to create *L. lactis* biosensors, the first one is based on the methionine auxotrophy of this bacterium and the second strategy is based on a cysteine-responsive promoter. The characterization of the biosensors showed their specific response to the presence of these amino acids. Subsequently, we applied the methionine biosensor to quantify the presence of methionine in bacterial supernatants of wild-type *L. lactis* that naturally secretes methionine to benchmark the performance of our biosensors. The methionine biosensor responded linearly to the amounts of methionine present in the bacterial supernatants, i.e., the increases in the biosensor cell densities were proportional to the amounts of methionine present in the supernatants. The biosensors developed in this study tackle the limitations of amino acid quantification and the selection of strains with secretion of amino acids. These biosensors may eventually be used for screening of engineered strains to increase methionine and cysteine production, and may facilitate the detection of these amino acids in complex food matrices.

## Introduction

Amino acids are attractive metabolites in industrial microbiology, and these compounds find application as artificial sweeteners, as flavoring agents, as feed additives or for pharmaceutical purposes (Hirasawa and Shimizu, 2016; Pinu et al., 2018). Recently, new engineering approaches toward the development of amino acid-producing microbial cells have been developed. For example, the first described glutamate-secreting bacterium *Corynebacterium glutamicum* has been used in new applications to increase the production of glutamate and lysine at a large-scale (Georgi et al., 2005). In addition, the secretion of amino acids by bacteria simplifies the extraction and purification processes. However, we need direct cellular production measurements, because it is otherwise difficult to select individual bacterial cells with enhanced production and secretion of amino acids, due to their diffusion into the environment where the bacteria grow.

*Lactococcus lactis*, the model LAB, which is used as a starter culture for cheese making plays an important role in flavor formation and the production of lactic acid (Smit et al., 2005). Besides their production as bulk biochemicals by fermentative procedures, amino acids are precursors of flavor compounds in dairy fermentations (Marin and Krämer, 2007; D’Este et al., 2018). In the proteolysis of casein, specific amino acids are responsible for producing the thiols, alcohols, esters and aldehydes responsible for a wide array of flavors (van Kranenburg et al., 2002). The final flavor of dairy products depends on the concentrations and ratios of different key aroma compounds. Based on cheese trials and sensory panels, the contribution of amino acids to flavor formation has been described (Centeno et al., 2002; De Palencia et al., 2004; Gutiérrez-Méndez et al., 2008). Sulfur aroma is enriched by the presence of methionine and cysteine. The compounds 3-methylbutanal, methanethiol, dimethyl sulfide (DMS), 2-Methylpropanol, and dimethyl trisulfide (DMTS) are all aroma compounds produced from methionine (Dias and Weimer, 1998). For instance, methanethiol is one of the main flavor chemicals responsible for the sulfur aroma in cheese, and it is associated with this desirable aroma found in good quality Cheddar (Seefeldt and Weimer, 2000; Singh et al., 2003).

Several engineering strategies using bacteria have resulted in amino acid-producing cells. However, besides the requirements of the strain-engineering methods, amino acid quantification and screening of producer strains are currently difficult tasks that depend on tedious sample preparation methods and analytical methods (Bertels et al., 2012). Biosensors are analytical tools that can be used for detection of a wide range of compounds. These devices offer an emerging technology for food industry, as an alternative to conventional analytical techniques. In essence, biosensors consist of metabolite-sensing elements and reporting elements (Lim et al., 2015).

Sensing a compound can be achieved by different biosensor systems. One biosensor type consists of a sensing element coupled to a reporter gene. For instance, the transcription-dependent biosensors are based on promoter activation/repression in response to the presence/absence of a molecule (Fernandez-López et al., 2015). A second biosensor type consists of a constitutively fluorescent biosensor, the growth of which depends on the product of interest. In this second type of biosensors, producer strains engineered to produce high-yields of a chemical compound can be identified by co-cultivation with an auxotrophic strain, i.e., the biosensor strain is auxotrophic for the desired compound. The latter biosensor type can be illustrated by a study where a lysine auxotrophic *E. coli* strain was used to determine the total content of lysine in different feed ingredients (Chalova et al., 2007). A plethora of biosensors have been developed over the past decades for biotechnological and medical applications (Close et al., 2009; Zhang and Keasling, 2011; Brutesco et al., 2017). A remaining challenge in biosensor engineering is to optimize its sensing properties in order to assess the effective cell factory production capacity (Siedler et al., 2017).

In the present study, we developed *L. lactis* biosensors to detect methionine and cysteine. The methionine biosensor is based on the auxotrophic nature of *L. lactis* for this amino acid. This GFP-marked growth-based biosensor is able to translate the concentration of methionine in a sample. In contrast to methionine, cysteine is not an essential amino acid for *L. lactis*. Thus, the cysteine biosensor development is based on a cysteine-responsive promoter fused to the green fluorescent protein (*gfp*) gene. We adapted the *L. lactis* requirements to increase the cysteine uptake and to activate the cysteine-responsive promoter. This strategy resulted in a fluorescence-based biosensor for the detection of cysteine. Furthermore, we applied the methionine biosensors to correlate the methionine concentration in bacterial supernatants. Thus, we demonstrated that biosensors developed in this work can be used for the quantification of methionine or cysteine.

## Materials and Methods

### Bacterial Strains and Growth Conditions

The bacterial strains used in this study are listed in Table 1. *L. lactis* cells were routinely grown as standing cultures at 30°C in M17 broth (Difco^TM^ BD, NJ, United States) or in chemically defined medium (CDM) (Goel et al., 2012), supplemented with glucose (GM17; Sigma-Aldrich, MO, United States) at a concentration of 0.5% (w/v). CDM contained 49.6 mM NaCl, 20.1 mM Na_2_HPO_4_, 20.2 mM KH_2_PO_4_, 9.7 μM (±)-α-lipoic acid, 2.10 μM D-pantothenic acid, 8.12 μM nicotinic acid, 0.41 μM biotin, 4.91 μM pyridoxal hydrochloride, 4.86 μM pyridoxine hydrochloride, 2.96 μM thiamine hydrochloride, 0.24 μM (NH_4_)_6_Mo_7_O_24_, 1.07 μM CoSO_4_, 1.20 μM CuSO_4_, 1.04 μM ZnSO_4_, 20.12 μM FeCl_3_, 1.46 mM L-alanine, 1.40 mM L-arginine, 0.61 mM L-asparagine, 1.03 mM L-aspartic acid, 0.35 mM L-cysteine, 0.66 mM L-glutamic acid, 0.66 mM L-glutamine, 0.39 mM glycine, 0.16 mM L-histidine, 0.63 mM L-isoleucine, 0.89 mM L-leucine, 1.02 mM L-lysine, 0.27 mM L-methionine, 0.39 mM L-phenylalanine, 3.58 mM L-proline, 1.64 mM L-serine, 0.57 mM L-threonine, 0.18 mM L-tryptophan, 2.76 mM L-tyrosine, and 0.73 mM L-valine.

**TABLE 1:** Strains and plasmids used in this study.

Strain	Description	References
***L. lactis***		
MG1363	Opp^+^, DtpT^+^, Dpp^+,^ Lac^–^, Prt^–^; plasmid-free derivative of *L. lactis* subsp. *cremoris* NCDO712.	Gasson, 1983
WTmet	Lac^–^, Prt^–^, Ery^r^, Opp^–^, DtpT^–^, Dpp^+^; AG500 derivative, carrying the vector pSEUDO:P*_*usp45*_-sfgfp(Bs).*	Hernandez-Valdes et al., 2020a
Δ*met*	*L. lactis* MG1363 Δ*met* deletion mutant.	Hernandez-Valdes et al., 2020c
WTcys	Ery^r^, MG1363 derivative, *llmg_pseudo10*:P*_*cys*_-gfp.*	This study
AG500	Opp^–^, DtpT^–^, Dpp^+^; MG1363 Δ*pepO*, Δ*dtpT*, Δ*opp.*	Hagting et al., 1994; Kunji et al., 1995
MGcys	Ery^r^, Opp^–^, DtpT^–^, Dpp^+^; AG500 derivative, *llmg_pseudo10*:P*_*cys*_-gfp.*	This study
SK11	Lac^+^, Prt^+^, *L. lactis* subsp. *cremoris*	Siezen et al., 2005
WW4	Lac^+^, Prt^+^, *L. lactis* subsp. *lactis* biovar. diacetylactis	Hernandez-Valdes et al., 2020b
NCDO176	Lac^+^, Prt^+^, *L. lactis* subsp. *lactis* biovar. diacetylactis	Bandell et al., 1998
*IPLA838*	*Lac+, Prt+, L. lactis subsp. lactis biovar. diacetylactis*	Cárcoba et al., 2000
*WT PrtP +*	*Cmr, PrtP +, MG1363 carrying the plasmid pNZ521.*	*This study*
Δ*codY*	*PrtP-, MG1363 codY deletion mutant*,	Den Hengst et al., 2005
Δ*rel*	*PrtP-, MG1363 rel deletion mutant.*	*A kind gift of Saulius Kulakauskas*
Δ*codY PrtP +*	*PrtP +, MG1363 codY deletion mutant, carrying the plasmid pNZ521*	*This study*
Δ*rel PrtP +*	*PrtP +, MG1363 rel deletion mutant, carrying the plasmid pNZ521*	*This study*
*MG PfbaA-gfp*	*Eryr, Opp–, DtpT–, Dpp+; AG500 derivative, llmg_pseudo10:PfbaA-gfp.*	*This study*
***E. coli***		
*DH5*α	*F– φ80lacZΔM15 Δ(lacZYA-argF)U169 recA1 endA1 hsdR17(rK–, mK+) phoA supE44 λ– thi-1 gyrA96 relA1*	*Laboratory stock*
*pNZ521*	*Cmr, DH5α carrying the pNZ521 plasmid*	*Laboratory stock*
***Plasmids***	***Description***	***References***
*pSEUDO-gfp*	*Eryr, integration vector, pSEUDO:sfgfp(Bs) derivative, carrying the gene coding for the green fluorescent protein (sfGFP).*	Pinto et al., 2011
pNZ521	Cm^r^, carrying the *prtPM* genes encoding for the proteinase PrtP	Meijer and Hugenholtz, 1997

The *E. coli* DH5α strain (Life Technologies, Gaithersburg, MD, United States) was used as the host for cloning and it was grown at 37°C in Luria-Bertani broth or Luria-Bertani agar 1.5% (w/v) (Difco^TM^ BD, NJ, United States). When appropriate, the culture medium was supplemented with 250 μg mL^–1^ erythromycin or 25 μg mL^–1^ chloramphenicol.

The CDM-met and CDM-cys media used in this study were prepared based on the chemically defined medium (CDM) recipe, but without L-methionine or L-cysteine, respectively.

M17 and LB-agar plates were prepared by adding agar 1.5% (w/v), and glucose (GM17) or lactose (LM17) to M17. When appropriate, the culture medium was supplemented with 5 μg mL^–1^ erythromycin or chloramphenicol (Sigma-Aldrich, MO, United States) for *L. lactis*.

For overnight cultures, flow cytometry analysis and plate-reader assays, *L. lactis* cells were grown in CDM with glucose 0.5% (w/v) and collected by centrifugation from exponential growth cultures (optical density of 0.4 at 600 nm) and washed three times with phosphate-buffered saline (PBS) solution (pH 7.2) containing: KH_2_PO_4_ 15.44 μM, NaCl 1.55 mM and Na_2_HPO_4_ 27.09 μM.

### Recombinant DNA Techniques and Oligonucleotides

Procedures for DNA manipulations (gel electrophoresis and transformation) were performed as described by Sambrook and Russell, 2001. PCRs were performed in an Eppendorf thermal cycler (Eppendorf, Hamburg, Germany) with *L. lactis* MG1363 chromosomal DNA as template, using Phusion polymerase (Thermo Fisher Scientific Inc., MA, United States). Oligonucleotides P1591_Fw (5′-CTAATACTCGAGACTCTGTCAGTAAAAAAGTGACAG-3′), P1591_Rv (5′-TTCAAAGCATGCCTTTTTGGTAAAGATAAA GAAGGGC-3′), PfbaA_Fw (5′-GGGTCGATCGAATTCGGTC CTCGGGATATG-3′) and PfbaA_Rv (5′- GACTTTGCAAGCTT GCATGCCTGCAGGTCG-3′) were purchased from Biolegio (Nijmegen, Netherlands). Plasmid DNA and PCR products were isolated and cleaned-up with a High-Pure plasmid isolation kit (Roche Applied Science, Mannheim, Germany), according to the protocol of the manufacturer. Colony PCR and subsequent sequencing (Macrogen, Amsterdam, Netherlands) was used to verify the constructs.

### Construction of *L. lactis* Strains

We used the *L. lactis* MG1363 and AG500 strains. All constructed strains are described in Table 1. To construct the vector pSEUDO:*P_*cys*_-gfp*, carrying the *cys* promoter of *L. lactis* MG1363, the promoter region was amplified by PCR using the P1591_Fw and P1591_Rv, using chromosomal DNA as template. The PCR fragment was cleaved with *Pae*I/*Xho*I enzymes and ligated to pSEUDO-gfp (Pinto et al., 2011). The vector pSEUDO:*P_*cys*_-gfp* was introduced in *L. lactis* MG1363 via electroporation (Holo and Nes, 1989). The vector was integrated into the silent *llmg_pseudo10* locus of *L. lactis* MG1363 by a single-crossover integration as described previously (Overkamp et al., 2013). Transformants were selected on M17-agar plates supplemented with sucrose, glucose and erythromycin 5 ug mL^–1^, yielding the *L. lactis* WTcys strain. The vector pSEUDO:*Pcys-gfp* was introduced by electroporation in *L. lactis* AG500 strain (a kind gift of Bert Poolman), resulting in the *L. lactis* MGcys strain.

To construct the plasmid pSEUDO:P*_*fbaA*_-gfp* carrying the *L. lactis fbaA* promoter upstream of the *gfp* gene, the *fbaA* promoter was amplified by PCR using the oligonucleotides PfbaA_Fw and PfbaA_Rv, and chromosomal DNA as template. The PCR fragment was digested with *Pae*I/*Xho*I and ligated to pSEUDO-gfp digested with the same enzymes. Plasmid pSEUDO:P*_*fbaA*_-gfp* were introduced in *L. lactis* AG500 as described above, yielding the *L. lactis* P*fbaA-gfp.*

To construct the WT PrtP + strain, the vector pNZ521 was isolated from *E. coli* DH5α pNZ521 using a High-Pure plasmid isolation kit (Roche Applied Science, Mannheim, Germany), according to the protocol of the manufacturer. The vector pNZ521 was introduced to *L. lactis* MG1363, *L. lactis* Δ*codY*, and *L. lactis* Δ*rel* by electroporation, yielding the WT PrtP +, Δ*codY* PrtP + and Δ*rel* PrtP + strains, respectively.

### Flow Cytometry

*Lactococcus lactis* cultures were grown overnight in CDM as described above, washed three times in PBS and transferred to fresh CDM-cys (containing 0.07 mM methionine), supplemented with increasing cysteine concentrations (0.1–2 mM). The cultures were incubated at 30°C and samples were taken at beginning of the stationary growth phase. The GFP-signal in all samples was recorded in 10,000 events (cells) and used for downstream analysis (named ungated events in the corresponding figures). GFP-signal measurements were obtained with a FACS Canto flow cytometer (BD Biosciences, CA, United States) using a 488 nm argon laser. A threshold for the FSC and SCC parameters was set (200 in both) in the FACS Canto flow cytometer (BD Biosciences, CA, United States) to remove all the events that do not correspond to cells. Raw data was collected using the FACSDiva Software 5.0.3 (BD Biosciences). And the FlowJo software was used for data analysis^1^.

### Growth and Fluorescence Measurements

Cultures of *L. lactis* were grown and prepared as described above. For fluorescence intensity measurements, *L. lactis* cells were diluted 1:20 in CDM. When testing the effect of varying concentrations of methionine or cysteine, CDM-met or CDM-cys were used, respectively, and supplemented with different amino acid concentrations (L-methionine 0.0004, 0.00097, 0.001963, 0.0039, 0.008, 0.016, 0.031, 0.063, 0.125, 0.15, 0.25, 0.5, 1, 2, 3, 4, 5, 10, 20 mM; L-cysteine 0, 0.05, 0.1, 0.2, 0.3, 0.4, 0.5, 0.6, 0.7, 0.8, 0.9, 0.95, 1 mM). The growth and fluorescence signals were measured in 0.2 mL cultures in 96-well micro-titer plates by using a micro-titer plate reader Varioskan (Thermo Fisher Scientific Inc., MA, United States). The optical density at 600 nm (OD_600_) and the GFP-signal with excitation at 485 nm and emission at 535 nm were measured. A measurement protocol in Varioskan was set to record the maximum value for fluorescence (RFU) in each well of the plate reader, and their corresponding optical density value at 600 nm (OD_600_). Both signals were corrected for the background values of the corresponding medium used (CDM) for growth.

The calculation used for resolving the relative GFP measurements (RFU/OD_600_) of the MGcys and WTcys cultures is depicted by the following equation:

$$\frac{G\,F\,P\,b\,i\,o\,s\,e\,n\,s\,o\,r-G\,F\,P\,m\,e\,d\,i\,u\,m}{O\,D\,b\,i\,o\,s\,e\,n\,s\,o\,r-O\,D\,m\,e\,d\,i\,u\,m}-\frac{G\,F\,P\,c\,o\,n\,t\,r\,o\,l-G\,F\,P\,m\,e\,d\,i\,u\,m}{O\,D\,c\,o\,n\,t\,r\,o\,l-O\,D\,m\,e\,d\,i\,u\,m}$$

Where GFP_biosensor_ and OD_biosensor_ are the fluorescence (RFU) and optical density values (OD_600_) of the *L. lactis* strain bearing the *cys* promoter fused to the *gfp* gene. GFP_medium_ and OD_medium_ are the fluorescence and optical density values of the growth medium (CDM-cys). The GFP_control_ and OD_control_ are the fluorescence and optical density values of the wild-type *L. lactis* strain. The maximum value of the fluorescence peak in each sample was considered as GFP value in all figures of this work and corrected with the equation mentioned above, yielding the relative fluorescent values (RFU/OD_600_).

### Fluorescence Microscopy

Washed cells were transferred to a solidified thin layer of growth medium with high-resolution agarose 1.5% (w/v) (Sigma-Aldrich, MO, United States). A standard microscope slide was prepared with a 65 μL Gene Frame AB-0577 (1.5 × 1.6 cm) (Thermo Fisher Scientific Inc., MA, United States). A 30 μL volume of heated CDM-agar was set in the middle of the frame and covered with another microscope slide to create a homogeneous surface after cooling. The upper microscope slide was removed and 1 μL of bacterial cells were spotted on the agar. The frame was sealed with a standard microscope coverslip.

Microscopy observations and time-lapse recordings were performed with a temperature-controlled (Cube and box incubation system Life Imaging Services) DeltaVision (Applied Precision, Washington, United States) IX7I microscope (Olympus, PA, United States), at 30°C. Images were obtained with a CoolSNAP HQ2 camera (Princeton Instruments, NJ, United States) at ×60 or ×100 magnification. 300-W xenon light source, bright-field objective and GFP filter set (filter from Chroma, excitation 470/40 nm and emission 525/50 nm). Snapshots in bright-field and GFP-channel were taken with 10% APLLC while LED light and a 0.05 s exposure for bright-field, or 100% xenon light and 0.8 s of exposure for GFP-signal detection. The raw data was stored using softWoRx 3.6.0 (Applied precision) and analyzed using ImageJ software (Schindelin et al., 2012).

### Quantification of Methionine

#### HPLC Assay

For sample preparation, each bacterial strain was inoculated in 10 mL of CDMcasein and grown at 30°C. Following growth, 5 mL of each culture was harvested by centrifugation at OD_600_ = 1.5. The supernatants were transferred into a clean tube, filtered through nitrocellulose Whatman filters (0.45 and 0.2 μm) and stored at 4°C for subsequent HPLC analysis.

Derivatization of standard methionine and samples with o-phthalaldehyde (OPA) reagent solution was set to automatically being carried out in the HPLC autosampler. Briefly, the derivatization was performed with a programmable automatic injector by mixing 1 μL of sample (or standard solution) with 2.5 μL of borate buffer pH = 10.4. After 0.2 min, 0.5 μL of OPA is added and mixed. Followed by mixing 32 μL of solvent A (10 mM Na_2_HPO_4_ and 10 mM Na_2_B_2_O_7_, pH 8), and the final injection of the whole mixture.

##### HPLC conditions

HPLC amino acid analysis was performed on an Agilent 1100 HPLC binary system (Agilent, Santa Clara, United States) equipped with an 1100 Fluorescence detector (FLD) and a Gemini C18 column (2 × 250 mm, 5 μm, Phenomenex, Torrance, United States). Borate buffer (0.4 M H_3_BO_3_) was used, and the mobile phases consisted of Solvent A (10 mM Na_2_HPO_4_ and 10 mM Na_2_B_2_O_7_, pH 8.2) and Solvent B (mixture of 45:45:10 acetonitrile/methanol/water). An aliquot of 1 μL derivatized sample (ad described above) was injected into the HPLC column equilibrated with Solvent A. The elution was carried out at a flow rate of 0.5 ml/min with the following program: from 0 to 0.5 min in 2% Solvent B, from 0.5 to 20 min gradient step to reach 57% Solvent B, from 20 to 20.1 min gradient step 57–100% solvent B, 20.1 to 23.5 min 100% solvent B, 23.5 to 23.6 min from 100 to 2% solvent B, and at 25 min ended.

The fluorescence detector (FLD) was set to Ex = 340 nm Em = 450 nm for the OPA derivatives. Quantifications of methionine were performed based on a five point calibration line between 5 and 500 μM. Data analysis was performed by using the Chemstation software to quantify methionine. The concentrations of methionine were obtained by measuring the FLD peak areas.

#### Biosensor Method

For quantification of methionine by using the WTmet biosensor, the bacterial supernatants were concentrated by freeze-drying and resuspended in fresh CDM-met. Next, the WTmet biosensor was grown in the CDM-met media (with the contents of the bacterial supernatant) in a 96-well plate. Cell densities (growth) values were obtained with measurements of the maximum optical density at 600 nm (OD_600_) in 96-well micro-titer plates by using a micro-titer plate reader Varioskan (Thermo Fisher Scientific Inc., MA, United States). The methionine concentrations in the samples were calculated by the interpolation method using the equation model of the WTmet curve (Supplementary Figure S1).

### Co-Cultivation Experiments

The CDMcasein medium used in the co-cultivation experiments was prepared based on the chemically defined medium recipe but without methionine (it contains the remaining 19 amino acids) and supplemented with casein according to Hammarsten 1% (w/v) (Merck & Co., NJ, United States) and glucose (Sigma-Aldrich, MO, United States) at a concentration of 0.5% (w/v).

Washed cells of both strains (WTmet and the PrtP + *L. lactis* strain) were adjusted to optical density of 0.5 at 600 nm and mixed in a 1:10 ratio (methionine-secreting PrtP + strain + : WTmet biosensor). The mixture of cells was used to perform time-lapse experiments or plate-reader assays.

### Statistics and Reproducibility

Statistical analyses were performed using Prism 6.01 (GraphPad software)^2^ and R v3.3.0. All experiments were repeated independently at least three times.

## Results

### A Growth-Based Biosensor for Methionine Detection

Lactococci are fastidious in nutrient requirements. For instance, most *L. lactis* strains are auxotrophic for several amino acids: isoleucine, leucine, valine, glutamic acid, histidine and methionine (Teusink et al., 2011; Adamberg et al., 2012). In spite of the fact that plants and most microorganisms are able to synthetize methionine *de novo, L. lactis* is auxotrophic for this amino acid (Seefeldt and Weimer, 2000). Methionine is an essential cellular compound due to its role as the universal N-terminal amino acid in protein synthesis and its participation in methylation reactions (Brosnan and Brosnan, 2006). In fact, the low methionine availability in milk is a limiting factor for growth of some lactic acid bacteria (Sperandio et al., 2005).

**FIGURE 1:**
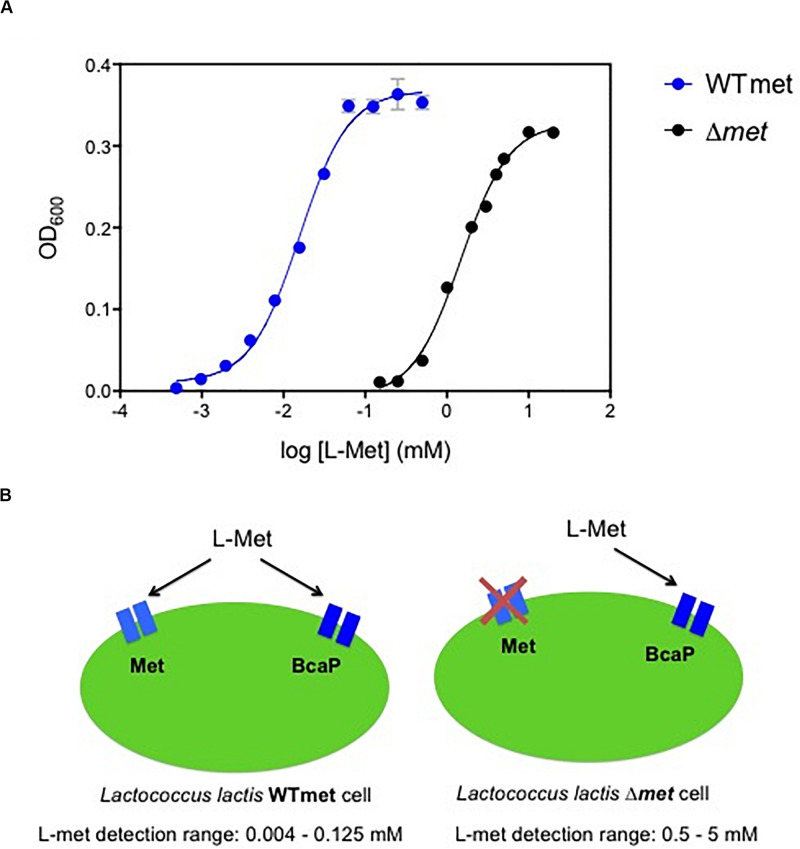
Methionine biosensors. **(A)** Dynamic range comparison of the biosensors for methionine detection. Overlapped dose-response curves of the methionine sensor WTmet and Δ*met* strains, blue and black lines, respectively. Growth measurements (optical cell density; OD_600_) were performed with CDM-met. The correlation between methionine concentrations [log (L-Met) mM] and the maximum optical cell density reached of bacterial cultures (OD_600_). Dots represent the average values of independent experiments (*n* = 3). Error bars represent standard deviation (SD) of the mean values of the three independent experiments. **(B)** Schematic representations of the methionine *L. lactis* biosensors, including the methionine detection range for each strain: WTmet imports methionine via a low-affinity permease BcaP and via the high-affinity transporter Met, and it allows detection of methionine concentrations in the range 0.004–0.125 mM; and Δ*met* exclusively imports methionine via the low-affinity permease BcaP, works well in the methionine concentration range 0.5–5 mM.

The auxotrophic nature of *L. lactis* for methionine makes it an attractive bacterial host to design growth-based biosensors to detect this amino acid. When methionine is available in the environment, it can be transported by two uptake systems that have been described previously: an ABC transporter (Met) and the branched-chain amino acid permease (BcaP) (Trip et al., 2013; Hernandez-Valdes et al., 2020c). We used a wild-type *L. lactis* (WTmet) strain and measured the culture cell densities, when growing in chemically defined medium (CDM-met) supplemented with different methionine concentrations (0.0004–20 mM). Figure 1A (blue line) shows the correlation between methionine concentration and optical cell density output. The WTmet growth is proportional to the amounts of methionine in CDM, in other words, the lower the methionine concentrations, the lower WTmet growth. This dose-curve, a S-shape sigmoidal curve, shows that WTmet provides a linear range of growth output in the range of approximately 0.004–0.125 mM (Supplementary Figure S1A,B). Figure 1B (left scheme) illustrates the two transporters involved in the methionine uptake by *L. lactis*. The Met transporter exclusively exports methionine, whereas BcaP preferably imports branched-chain amino acids (valine, leucine, isoleucine) and to a lesser extent methionine (Trip et al., 2013). Based on the affinity of these transporters to methionine, the deletion of the Met transporter might results in ineffective methionine uptake, and indirectly affects the *L. lactis* methionine requirement. Thus, we hypothesized that a change in the methionine affinity might result in a change of the dynamic range of the methionine-dependent growth of the *L. lactis* biosensor.

### Tuning the Dynamic Range of the Methionine Biosensor

Next, we used a *L. lactis met* deletion mutant (**Δ***met*) strain and measured the culture cell densities when it grows in chemically defined medium (CDM-met) supplemented with different methionine concentrations (0.125–20 mM). Figure 1A (black line) shows the correlation between methionine concentration and optical cell density output. This dose-curve is a S-shape sigmoidal curve as well (Supplementary Figure S2). Indeed, we confirm that the *met* deletion results in a new dynamic range. The *L. lactis* **Δ***met* growth requires higher methionine concentrations to grow compared to the wild type (WTmet). This dose-curve shows that **Δ***met* provides a linear range of growth output in the range of approximately 0.5–5 mM. Figure 1B (right scheme) illustrates the only available path for methionine uptake in this strain; the higher methionine requirements in this strain are explained by the low affinity of BcaP for methionine and its competition with the branched-chain amino acids to be taken up via this transporter (Hernandez-Valdes et al., 2020c).

The modulation of the affinity of the transporter for the essential compound in an auxotrophic biosensor for the compound can contribute to the development of altered sensitivities. This effect on sensitivity is depicted in Figure 1A, where a comparison of the dose-curves between our methionine biosensors is shown. The dynamic range is an important indicator for fine-tuning biosensors. The deletion of one uptake pathway tunes the dynamic range by reducing the methionine affinity.

### A Transcription-Based Biosensor for Cysteine Detection

**FIGURE 2:**
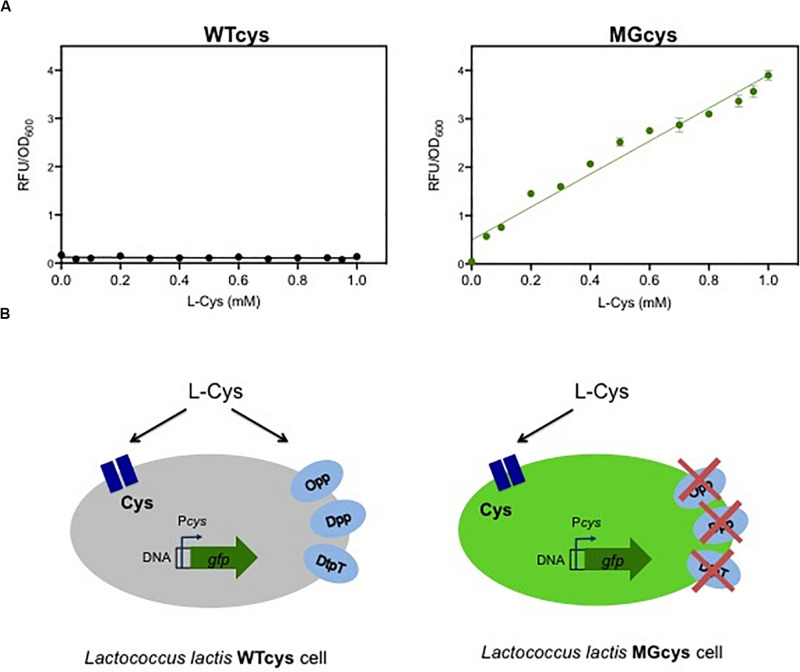
A responsive promoter-based cysteine biosensor. **(A)** Dose-response curves of the cysteine biosensor in the WTcys and MGcys strains. The correlation between cysteine concentrations (L-Cys; mM) and the relative fluorescence signals to the corresponding optical cell density of bacterial cultures (OD_600_). Dots represent the average values of independent experiments (*n* = 3). Error bars represent standard deviation (SD) of the mean values of the three independent experiments. **(B)** Schematic representation of the cysteine uptake in *L. lactis* and the *cys* promoter activation in two genetic backgrounds (WTcys and MGcys). The WTcys strain bearing the oligopeptide transport systems (Opp, Dpp, and DtpT) shows very low activation levels of the *cys* promoter (left cell; shown in gray). In contrast, the MGcys strain that lacks the oligopeptide transport systems activates the *cys* promoter to obtain cysteine (right cell; shown in green).

In contrast to the essentiality of methionine, *L. lactis* is able to synthesize cysteine, using serine or methionine as substrates. In addition, *L. lactis* might take up extracellular cysteine. The genome of *L. lactis* MG1363 encodes a putative single Cys-transporter in the *cys* operon, composed of three genes: *llmg_1593* encodes an amino acid ABC transporter substrate binding protein, *llmg_1591* encodes the permease protein and *llmg_1590* encodes the ATP binding protein. To visualize the expression of the Cys*-*transporter at different cysteine concentrations, we fused the *cys* promoter to a gene encoding for a green fluorescent protein (*gfp*). The resulting strain WTcys shows that the Cys transporter is not expressed in the wild-type *L. lactis* MG1363, even at very low concentrations of cysteine in the medium (Figure 2A; left plot). Since no other putative cysteine transporter is reported for this bacterium, we investigated whether cysteine can be taken up by the cells via a more general mechanism, such as the oligopeptide or peptide transport systems (Opp, Dpp, DtpT). Next, we used a *L. lactis* lacking these peptide transport systems (named MG), to evaluate the *cys* expression in this genetic background. The resulting strain MGcys was again used to visualize the expression of the Cys*-*transporter at different cysteine concentrations. This strain shows that the Cys-transporter is expressed in a concentration dependent way, i.e., at higher cysteine concentrations the higher expression levels of the *cys* operon (Figure 2B; right plot). In addition, this dose-curve follows a linear regression model (Supplementary Figure S3). This finding implies that cysteine can be imported into the *L. lactis* cell via one or more peptide uptake systems in the wild type, and thus it results in very low levels Cys-expression levels when these alternative uptake systems are functional (see Figure 2B).

### Genetic Mechanism Involved in Cysteine Detection

**FIGURE 3:**
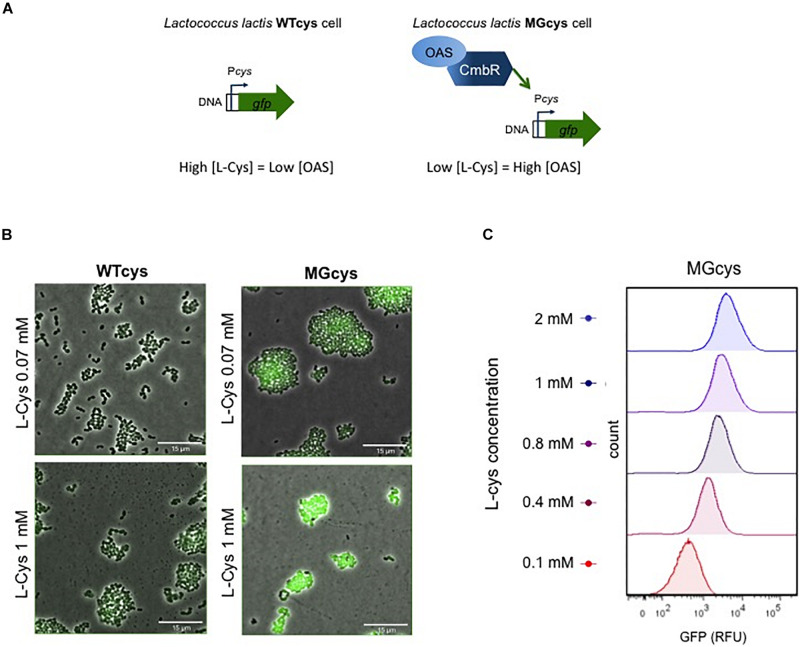
Factors involved in the activation of the *cys* promoter. **(A)** The *cys* promoter is regulated by the CmbR transcription factor. In the WTcys cell (left), cysteine levels are saturated and the expression of the Cys transporter is not required. In contrast, in the MGcys strain the low levels of cysteine trigger the formation of O-acetyl serine (OAS), which co-induces the *cys* promoter via the CmbR regulator. **(B)** Fluorescence microscope pictures of WTcys and MGcys strains grown in CDM-cys and supplemented with either low (0.07 mM) or high (1 mM) cysteine. Snapshots of time-lapse experiments are shown. The GFP-marked WTmet sensor cells are cultivated with different lactococcal strains. Overlays of fluorescence-channel and bright-field are shown. Overlays of fluorescence-channel and bright-field are shown. **(C)** Single-cell fluorescence measurements of the MGcys strain by flow cytometry, in the presence of increasing concentrations of cysteine (red to blue, 0.1–2 mM). Fluorescence measurements were taken at the beginning of stationary growth phase. 10,000 ungated events for each sample are shown.

As mentioned above, *L. lactis* also can use serine as a substrate for cysteine biosynthesis. Serine is converted into O-acetyl-serine (OAS) by a serine O-acetyl transferase CysE, and OAS is subsequently converted into cysteine by activity of cysteine synthase (Supplementary Figure S4; Fernández et al., 2002; Sperandio et al., 2005). This biosynthetic pathway is involved in the *cys* promoter activation. Previous studies reveal that the CmbR transcription factor regulator activates the *cys* promoter (Fernández et al., 2002; Guédon and Martin-Verstraete, 2006). In addition, the binding of CmbR to its target genes is co-induced by the presence of OAS (Golic et al., 2005). Based on this evidence, we explain the activation of the *cys* promoter in the two genetic backgrounds we used. Figure 3A illustrates a model where in the wild type strain (WTcys) the cysteine requirements are satisfied via the peptide uptake systems, resulting in low usage of the biosynthetic pathway and thus low levels of OAS. In contrast, in the MGcys strain, lacking the extracellular uptake of cysteine, the biosynthetic pathway to synthesize cysteine from serine is used, resulting in high levels of OAS and thus, high levels of the Cys-transporter expression.

Thus, we obtained a cysteine biosensor, which provides a linear response in the cysteine concentration range 0.07–1 mM. Next, we examined *L. lactis* WTcys and MGcys cells using fluorescence microscopy in CDM (containing 0.07 mM methionine) supplemented with low (0.07 mM) and high (1 mM) cysteine. Figure 3B shows that at low cysteine concentrations, the activation of the *cys* promoter is low in both strains, but MGcys shows higher levels than WTcys. In contrast, at high cysteine concentrations, only the MGcys strain shows very high levels of *cys* expression (see Supplementary Movies S1–S4). Moreover, we performed single-cell fluorescence measurements of the MGcys strain in the presence of different cysteine concentrations by flow cytometry. Cells grown in CDM with increasing cysteine concentrations show proportional increasing levels of GFP expression (Figure 3C). Therefore, despite the background fluorescence intensity levels that the MGcys strain shows at low cysteine concentrations (0.1 mM), the high fluorescence intensity levels it reaches at high cysteine concentrations (1 mM), results in clearly separable cell populations. This result indicates that this sensor is suitable to correlate cysteine concentrations with fluorescence outputs.

In contrast to methionine, cysteine is toxic at low concentrations and therefore, its concentration is stringently regulated in bacteria (Guédon and Martin-Verstraete, 2006; Takumi and Nonaka, 2016). In order to discard the possibility that this toxic effect affects our GFP measurements at high cysteine concentrations, we constructed a transcriptional fusion with the promoter P*fbaA* (MG P*fbaA-gfp* strain). The *fbaA* gene encodes the fructose-bisphosphate aldolase, a key enzyme in the glycolysis pathway, i.e., it has a housekeeping role in metabolism (Shams et al., 2014). We performed single-cell GFP measurements by flow cytometry when the strains are growing in the presence of different cysteine concentrations (Supplementary Figure S5). Accordingly, we observe a homogenous GFP expression in the MG P*fbaA-gfp* and WTcys strains when growing in the presence of different cysteine concentrations, and a shift in the GFP expression levels of the MGcys strain. Furthermore, the effect of cysteine on *L. lactis* growth is observed in growth curves (Supplementary Figure S6), cysteine retards the bacterial growth at higher concentrations than 0.1 mM. Importantly, the growth curves and the corresponding GFP expression measurements corroborate that the cysteine effect on growth does not affect the cysteine-induction of the MGcys strain.

Two drawbacks for the MGcys biosensor are the toxic effect of cysteine and the fact that methionine and serine can be converted into cysteine. Firstly, the cysteine toxicity at low concentrations limits the possibility to use a growth-based sensor to detect cysteine (obtaining an auxotrophic *L. lactis* for cysteine). Thus, a transcription-based sensor is a suitable alternative. Secondly, *L. lactis* can use methionine or serine as substrates for cysteine biosynthesis. Indeed, at high methionine concentrations the *cys* promoter is not activated in any of the two genetic backgrounds (WTcys nor MGcys; Supplementary Figure S6). To obtain an auxotrophic *L. lactis* strain for cysteine, gene deletions in the pathways of methionine and serine are required. However, a disruption in the serine pathway would affect the mechanism of transcriptional sensing in our MGcys biosensor.

### Benchmarking the Methionine Biosensor

**FIGURE 4:**
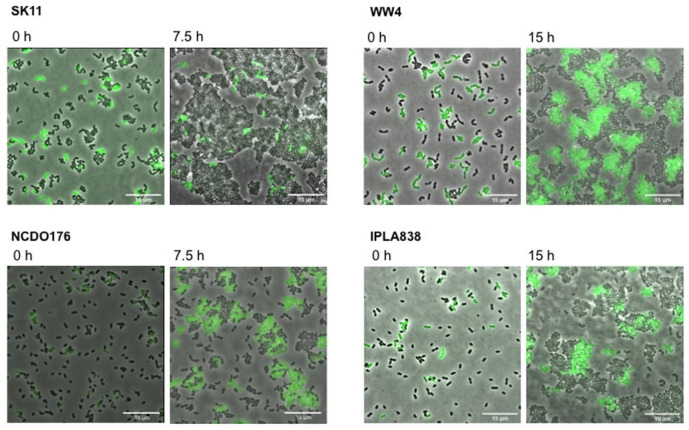
Methionine secretion by *L. lactis* strains is detected with the methionine biosensor. Positive amino acid-secreting *L. lactis* strains WW4, IPLA838 and WW4, and negative amino acid-secreting strain SK11 are co-cultivated with the GFP-marked WTmet biosensor. The WTmet biosensor lacks the oligopeptide peptide transport systems and therefore, it is unable to grow in CDMcasein-met (supplemented with casein 1% (w/v) and 19 amino acids, except methionine), unless the amino acid producer strain secretes the essential amino acid methionine. Snapshots of time-lapse experiments are shown at inoculation time (0 h) and at the beginning of the stationary growth phase (7.5 and 15 h). Overlays of fluorescence-channel and bright-field are shown.

We further characterized the methionine biosensor. We used the *L. lactis* GFP-marked strain (WTmet; see Table 1), which exhibits a constitutive GFP expression throughout the culture, to test the performance of the constructed biosensor by monitoring its growth and/or fluorescence when it is exposed to the presence of samples containing methionine. The secretion of several amino acids including methionine by certain lactococcal strains has been reported in a previous study (Hernandez-Valdes et al., 2020b). Thus, we used the *L. lactis* WW4, NCDO176, and IPLA838 as positive methionine-secreting strains to test our WTmet sensor. Figure 4 shows our strategy based on co-cultivation of the GFP-marked WTmet biosensor and a methionine producer, grown in CDMcasein supplemented with 19 amino acids, except methionine. The methionine producers are proteinase positive (PrtP +) strains that are able to grow in medium containing casein as nitrogen source. In contrast, the WTmet sensor lacks the PrtP enzyme and the peptide transporters, and thus it is fully dependent on the methionine uptake via the BcaP- and Met-transporters. Consequently, the growth observation of WTmet cells by GFP expression is used as an indication of methionine secretion by the wild-type strains. Thus, the lower the amounts of methionine, the lower the amount of WTmet cells (GFP + cells). Accordingly, Figure 4 shows that the growth of the WTmet biosensor is highly promoted by co-cultivation with the WW4, NCDO176 and IPLA838 strains (positive methionine-secreting strains), whereas the SK11 strain, which indeed is used as a control (it is a negative methionine-secreting strain), does not promote the WTmet growth (see Supplementary Movies S5–S8).

### Methionine Secretion Is Enhanced by *codY* and *rel* Mutants

In general, *L. lactis* employs two global nitrogen regulators to regulate amino acid uptake: CodY and Rel. Firstly, CodY is a transcription factor that represses the expression of amino acid transporters when amino acids are abundant (Guédon et al., 2001). For instance, CodY represses the *bcaP* expression (Den Hengst et al., 2006). Secondly, Rel is a bifunctional protein that can both synthesize and degrade phosphorylated purine-derived alarmones (p)ppGpp (Potrykus and Cashel, 2008). In this manner, Rel can activate the so-called stringent response, which is a general stress response triggered by nutrient stress, such as amino acid starvation (Geiger and Wolz, 2014; Fang and Bauer, 2018). We therefore hypothesize that mutations in these regulators affect the secretion of amino acids.

**FIGURE 5:**
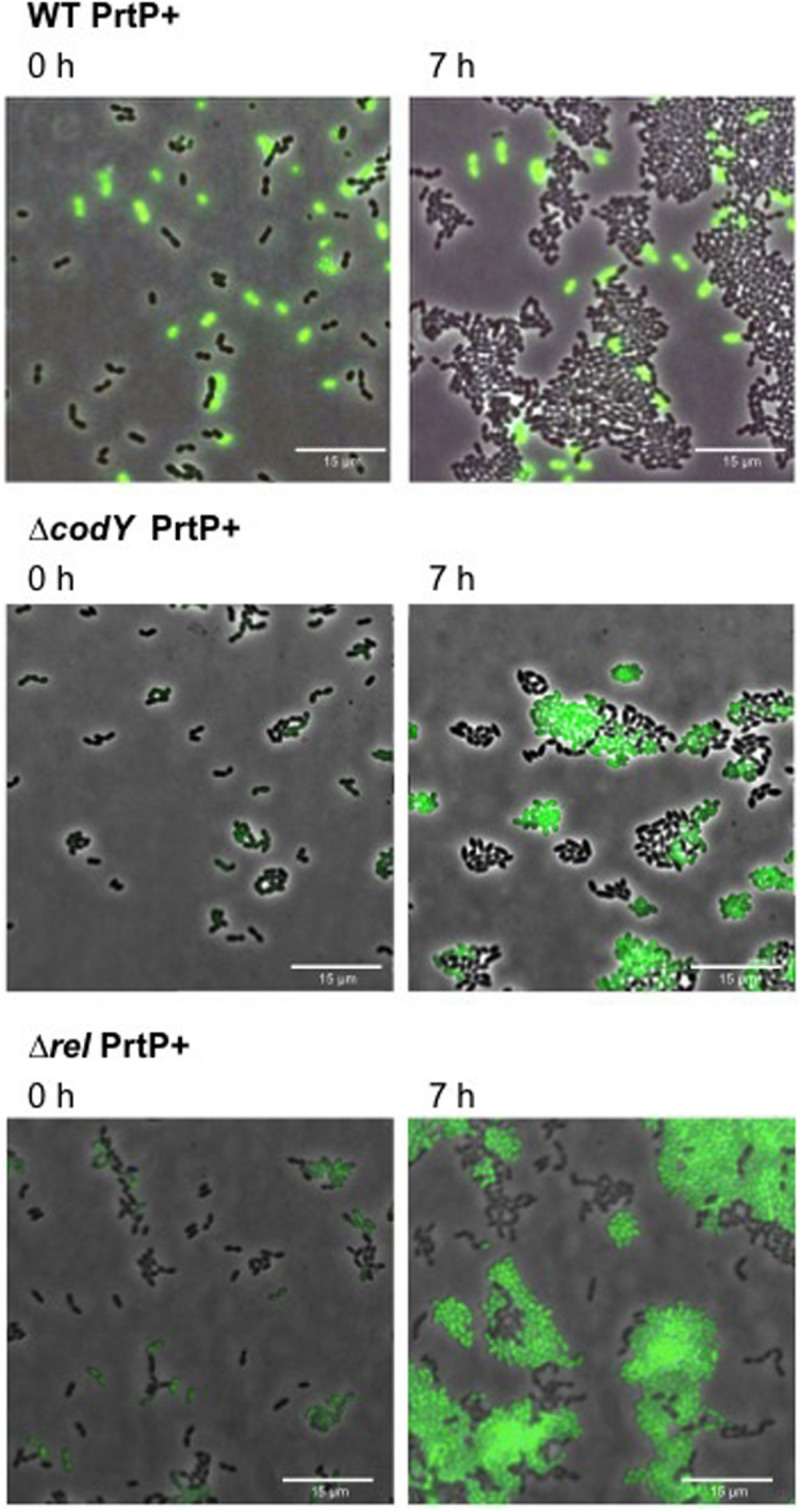
Methionine secretion by *L. lactis codY* and *rel* deletion mutants. Snapshots of time-lapse experiments are shown. The GFP-marked WTmet sensor cells are co-cultivated with different *L. lactis* strains (WT, Δ*codY*, and Δ*rel*) and grown in CDMcasein-met (supplemented with casein 1% (w/v) and 19 amino acids, except methionine). Overlays of fluorescence-channel and bright-field are shown.

To investigate the effect of *codY* and *rel* deletion on methionine secretion, we constructed the proteinase positive strains: WT PrtP +,**Δ***codY* PrtP +, **Δ***rel* PrtP +. Thus, these strains are able to grow on CDMcasein. We evaluated the methionine secretion by using our WTmet biosensor (GFP +). To this end, each PrtP + strain is co-cultivated with the WTmet biosensor in CDMcasein-met. Figure 5 shows that the deletion of *codY* and *rel* promotes the growth of the WTmet biosensor, compared to the wild type, which does not promote the biosensor growth (see Supplementary Movies S9–S11). These results indicate that **Δ***codY* PrtP + and **Δ***rel* PrtP + are able to secrete methionine. Remarkably, the deletion of *rel* results highly promotes the growth of the WTmet biosensor.

### Correlation Between Methionine Concentrations and Biosensor Output

To further confirm the application of the WTmet biosensor, we quantified the methionine concentration present in bacterial supernatants. We collected, filtered and concentrated supernatants of methionine-secreting bacterial cultures (see section “Materials and Methods”). The WTmet was grown in CDM-met supplemented with the methionine-containing bacterial supernatant. The methionine concentration was calculated by the interpolation method using the WTmet dose-curve, using the cell density values of the WTmet in the presence of each supernatant (Supplementary Figure S1B). Moreover, we also quantified the methionine content in the samples by an HPCL assay (see section “Materials and Methods”) to evaluate the biosensor performance. Table 2 confirms that the *rel* and *codY* deletion mutants are able to secrete methionine compared to the wild type (WT PrtP +). In addition, the methionine values are consistent with the fluorescence microscopy observations in Figures 4, 5, i.e., high methionine levels results in high number of WTmet cells. Remarkably, our WTmet biosensor provides similar concentration values to the quantification by the HPLC assay. Taken together, these results suggest that the WTmet biosensor can be used to semi-quantitatively detect methionine.

**TABLE 2:** Methionine quantifications.

	WTmet Biosensor		HPLC assay
		
*Sample*	*Cell density*	*L-met (mM)*	*L-met (mM)*
***Controls***			
Medium	ND	ND	ND
SK11	ND	ND	ND
L-met 0.06 mM	0.300 ± 0.020	0.043 ± 0.005	0.068 ± 0.001
WT PrtP +	ND	ND	ND
***Supernatants***			
WW4	0.297 ± 0.006	0.041 ± 0.005	0.052 ± 0.001
IPLA838	ND	ND	0.003 ± 0.001
NCDO176	0.173 ± 0.022	0.014 ± 0.001	0.014 ± 0.002
*codY* PrtP +	0.110 ± 0.001	0.008 ± 0.001	0.004 ± 0.002
*rel* PrtP +	0.140 ± 0.036	0.011 ± 0.001	0.009 ± 0.001

*Values indicate concentration (μM). After the strains were grown in CDMcasein-met medium, the concentrations of methionine in the culture supernatants were measured using a HPLC assay coupled with fluorescence detection and with the WTmet biosensor. Strains with positive (WW4, IPLA838, NCDO176) and negative (SK11) methionine secretion capacity are shown. In addition, two elution samples of the NCDO176 (E1 and E2) were also quantified. Positive (standard methionine solution containing L-met 0.06 mM) and negative (CDMcasein-met) controls are shown. Quantification below the detection limit of the HPLC assay (<0.001 mM) and WTmet biosensor (<0.004 mM) are indicated as ND. Quantifications were performed in duplicates, and average values are shown. Error is shown as SD.*

## Discussion

Methionine and cysteine are relevant amino acids for the food industry. These amino acids are a source of sulfur, and dietary essential for humans (Fukagawa, 2006). Sulfur is a major inorganic element, essential to the entire biological kingdom because of its incorporation into many biomolecules e.g., proteins and vitamins (Parcell, 2002). In dairy fermentations, these sulfur-containing amino acids participate in the flavor and aroma formation of products. The volatile sulfur compounds found in cheeses originate from methionine or cysteine (Dias and Weimer, 1998; Guédon and Martin-Verstraete, 2006). For instance, methanethiol is considered one important component of Cheddar cheese aroma (Singh et al., 2003). Moreover, the food industry is interested in creating synthetic flavors and aromas by combination of chemical compounds. As food additives, the meat-related flavor and sulfur aroma of foods is due to the presence of methionine and cysteine (McGorrin, 2011; Yang et al., 2015). For instance, methionine is used to enhance the soft flavor of potatoes (Di et al., 2003), and a combination of cysteine or methionine with reducing sugars creates a caramel smell (Zhang et al., 2018).

Besides their role in food industry, these sulfur amino acids play relevant biological roles in cell metabolism (Parcell, 2002). Methionine (N-formylmethionine) is the initiation amino acid in the synthesis of proteins in bacteria and cysteine is important in protein structure because of its ability to form inter- and intra-chain disulfide bonds with other cysteine residues (Brosnan and Brosnan, 2006).

In this work, we developed *L. lactis* biosensors to facilitate the detection of methionine and cysteine, produced and secreted by wild-type *L. lactis*. The identification of strains with improved production and secretion of these amino acids by bacteria is laborious because it requires tedious sample preparations methods. The construction of the cysteine biosensor is a transcription-based biosensor, and consists of a cysteine-responsive *cys* promoter fused to the green fluorescent protein (*gfp*) gene. We increased the cysteine requirements of *L. lactis* to activate the *cys* promoter and obtained the MGcys biosensor that responds to the presence of cysteine in a concentration range of 0–1 mM. One limitation of the cysteine biosensor is the toxicity of cysteine, which would cause a detection limit even in an auxotrophic *L. lactis* for cysteine. The cysteine toxicity is caused by the thiol (-SH) group, which makes cysteine a highly reactive compound (Guédon and Martin-Verstraete, 2006). A second limitation is the fact that *L. lactis* can convert both methionine and serine into cysteine. Thus, the sensor is functional when the methionine concentrations are very low (0.07 mM). However, in contrast to cysteine, methionine is an essential amino acid for *L. lactis*, and a low methionine concentration in the growth medium results in low cell densities. Therefore, future work is required to increase the bacterial tolerance to high concentrations of cysteine.

The construction of the methionine biosensor is based on the methionine auxotrophy of *L. lactis*. This GFP-marked growth-based biosensor (WTmet) is able to translate the concentration of methionine into growth readouts. The functionality of the WTmet biosensor was confirmed by observing its growth in co-cultivation with methionine-secreting strains. In addition, we reveal that deletion of *codY* and *rel* result in methionine secretion, since both regulators are involved in nitrogen regulation. An unbalance in the amino acid uptake might result in methionine overflow by this bacterium. A further study to investigate the molecular mechanism involved in methionine overflow by *L. lactis* is suggested.

Next, we benchmarked the methionine biosensor using the methionine-secreting bacteria and correlated its growth with the methionine concentration. Our data suggest that the WTmet biosensor can be applied to detect and quantify methionine in bacterial supernatants, since it provides similar results to the methionine quantification by a HPLC assay. Moreover, we tuned the dynamic range of the methionine biosensor by affecting the bacterial uptake of this amino acid. The biosensor concentration range 0.004–0.125 mM (WTmet) increased to the concentration range 0.5–5 mM (Δ*met*). The selection of biosensor depends on the concentration range in the target sample. For instance, in this work we demonstrate the application of our bacterial biosensor to detect and quantify methionine (produced and secreted) by wild-type *L. lactis*. All the methionine-secreting strains produce methionine in concentrations within the WTmet response range. Yet, methionine production and secretion by other bacteria or at large-scale might result in higher methionine concentrations, and thus these samples require biosensors with an increased dynamic range to quantify methionine.

## Data Availability Statement

All datasets presented in this study are included in the article/Supplementary Material.

## Author Contributions

JH-V and OK conceived the study and wrote the manuscript. JH-V designed and carried out the experiments for the methionine biosensors. MD carried out the experiments for the cysteine biosensors. JH performed the HPLC assay for methionine quantifications. OK provided the supervision. All authors discussed the results and commented on the manuscript.

## Conflict of Interest

The authors declare that the research was conducted in the absence of any commercial or financial relationships that could be construed as a potential conflict of interest.

## Footnotes

1 https://www.flowjo.com/

2 https://www.graphpad.com/
