# Supplementary material for: Development of Lactococcus lactis Biosensors for Detection of Sulfur-Containing Amino Acids
Source: Front Microbiol. 2020 Jul 15;11:1654. doi: 10.3389/fmicb.2020.01654 (PMC7375092; doi:10.3389/fmicb.2020.01654)
Supplement: Supplementary file 1 [file Data_Sheet_1.pdf]

Supplementary Material

1 Supplementary Figures

A

		OD600		
L-met (mM)	log [L-met]	Exp1	Exp2	Exp3
0.5000	-0.30	0.348	0.349	0.363
0.2500	-0.60	0.342	0.370	0.378
0.1250	-0.90	0.339	0.350	0.356
0.0625	-1.20	0.344	0.345	0.358
0.0313	-1.51	0.263	0.271	0.263
0.0156	-1.81	0.178	0.172	0.177
0.0078	-2.11	0.107	0.111	0.115
0.0039	-2.41	0.061	0.065	0.060
0.0020	-2.71	0.030	0.030	0.033
0.0010	-3.01	0.014	0.017	0.013
0.0005	-3.31	0.004	0.003	0.004

B

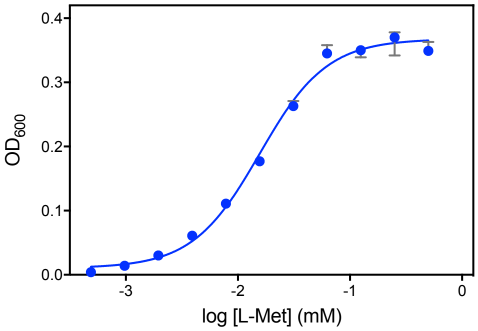

Y=Bottom + (Top-Bottom)/(1+10^((LogEC50-X)*HillSlope))

Y = 0.01029 + (0.3677 - 0.01029)/(1+10^((-1.805-X)*1.45))

R squared = 0.9931

C

	OD600				
Sample	Exp1	Exp2	Exp3	Average values log [L-met] interpolated	Average values [L-met] interpolated
WW4	0.30	0.29	0.30	-1.388	0.041
IPLA838	ND	ND	ND	ND	ND
NCDO176	0.16	0.20	0.16	-1.860	0.014
Medium	ND	ND	ND	ND	ND
SK11	ND	ND	ND	ND	ND
L-met 0.06mM	0.30	0.32	0.28	-1.370	0.043
WT PrtP+	ND	ND	ND	ND	ND
codY PrtP+	0.10	0.11	0.12	-2.089	0.008
rel PrtP+	0.10	0.15	0.17	-1.974	0.011

Supplementary Figure 1. A growth-based methionine biosensor. (A) Cell density measurements (OD_{600}) of WTmet strain at different methionine concentrations. Growth measurements (optical cell density; OD_{600}) were performed with CDM-met, supplemented with methionine at different concentrations (0.0005 – 0.5 mM). The maximum optical densities reached of bacterial cultures, in three independent experiments are shown (Exp1, Exp2, Exp3). **(B)** Dose-response curve of the methionine sensor WTmet strain obtained with the growth measurements shown in **(A)**, using the log [L-Met] (*x*-axis) and OD_{600} values (*y*-axis). The correlation between methionine concentrations and the cell density of bacterial cultures follows a symmetrical sigmoidal shape, and the equation of this model (Sigmoidal 4PL) is indicated. Dots represent the average values of independent experiments (n=3). Error bars represent standard deviation (SD) of the mean values of the three independent experiments. **(C)** Calculation of the methionine concentration in bacterial supernatants. The maximum optical densities reached of WTmet bacterial cultures, in three independent experiments are shown (Exp1, Exp2, Exp3). The average values (green column) were obtained by interpolation of the average values of the cell density, using the equation indicated in **(B)**. The lack of growth of the WTmet strain is indicated as non-detected (ND).

A

		OD_{600}		
L-met (mM)	log [L-met]	Exp1	Exp2	Exp3
0.15	-0.82	0.009	0.010	0.014
0.25	-0.60	0.015	0.012	0.009
0.50	-0.30	0.038	0.036	0.038
1.00	0.00	0.127	0.125	0.128
2.00	0.30	0.201	0.202	0.199
3.00	0.48	0.226	0.225	0.227
4.00	0.60	0.265	0.262	0.268
5.00	0.70	0.287	0.287	0.279
10.00	1.00	0.317	0.315	0.319
20.00	1.30	0.310	0.317	0.322

B

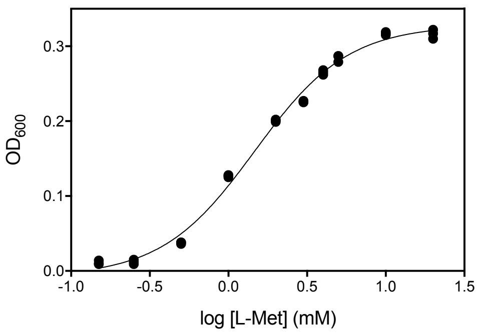

Y=Bottom + (Top-Bottom)/(1+10^((LogEC50-X)*HillSlope))

Y = -0.007719 + (0.3283-(-0.007719))/(1+10^((0.1681-X)*1.464))

R squared = 0.9931

Supplementary Figure 2. A methionine biosensor with increased target concentration. (A) Cell density measurements (OD_{600}) of the Δ*met* strain at different methionine concentrations. Growth measurements (optical cell density; OD_{600}) were performed with CDM-met, supplemented with methionine at different concentrations (0.15 – 20 mM). The maximum optical densities reached of bacterial cultures, in three independent experiments are shown (Exp1, Exp2, Exp3). **(B)** Dose-response curve of the methionine sensor Δ*met* strain obtained with the growth measurements shown in **(A)**, using the log [L-Met] (*x*-axis) and OD_{600} values (*y*-axis). The correlation between methionine concentrations and the cell density of bacterial cultures follows a symmetrical sigmoidal shape, and the equation of this model (Sigmoidal 4PL) is indicated. Dots represent the average values of independent experiments (n=3). Error bars represent standard deviation (SD) of the mean values of the three independent experiments.

A

WTcys

L-cys (mM)	RFU/OD600 Exp1	Exp2	Exp3
0.00	0.160	0.120	0.220
0.05	0.050	0.080	0.120
0.10	0.080	0.110	0.110
0.20	0.110	0.130	0.210
0.30	0.110	0.060	0.120
0.40	0.070	0.120	0.130
0.50	0.080	0.130	0.110
0.60	0.110	0.110	0.160
0.70	0.050	0.110	0.100
0.80	0.120	0.050	0.160
0.9	0.110	0.160	0.061
0.95	0.020	0.080	0.120
1	0.080	0.130	0.190

MGcys

L-cys (mM)	RFU/OD600 Exp1	Exp2	Exp3
0.00	0.050	0.110	0.000
0.05	0.550	0.630	0.540
0.10	0.750	0.800	0.720
0.20	1.450	1.420	1.490
0.30	1.650	1.550	1.600
0.40	2.150	2.050	2.010
0.50	2.450	2.500	2.610
0.60	2.750	2.800	2.720
0.70	3.000	2.900	2.720
0.80	3.110	3.150	3.040
0.9	3.250	3.350	3.500
0.95	3.700	3.450	3.550
1	3.800	3.900	4.000

B

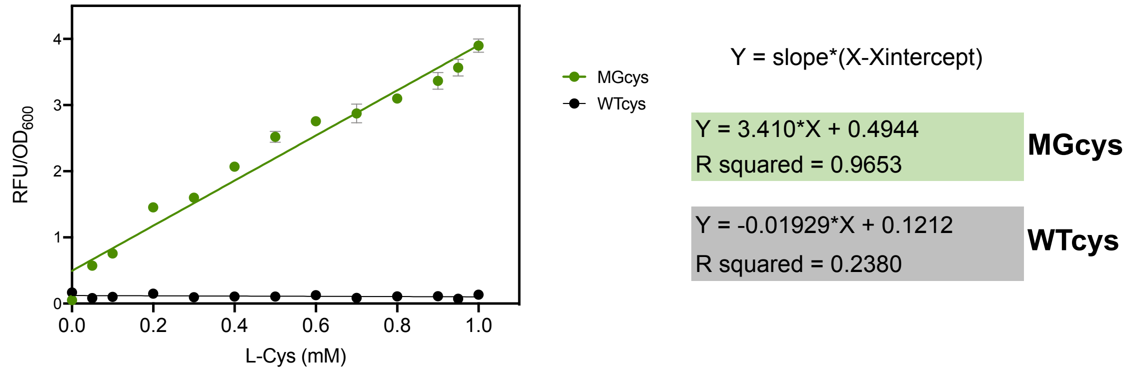

Supplementary Figure 3. A fluorescence-based biosensor to detect cysteine. (A) Cell density measurements (OD_{600}) of WTcys and MGcys strains at different cysteine concentrations, left and right, respectively. Growth measurements (optical cell density; OD_{600}) were performed with CDM-cys (containing 0.07mM methionine), supplemented with cysteine at different concentrations (0 – 1 mM). The maximum optical densities reached of bacterial cultures, in three independent experiments are shown (Exp1, Exp2, Exp3). **(B)** Dose-response curve of the cysteine sensor WTcys and MGcys strains obtained with the growth measurements shown in **(A)**, using the log [L-Cys] (*x*-axis) and OD_{600} values (*y*-axis). The correlations between cysteine concentrations and the cell density of bacterial cultures follow a linear regression, and the equations of these models are indicated. Dots represent the average values of independent experiments (n=3). Error bars represent standard deviation (SD) of the mean values of the three independent experiments.

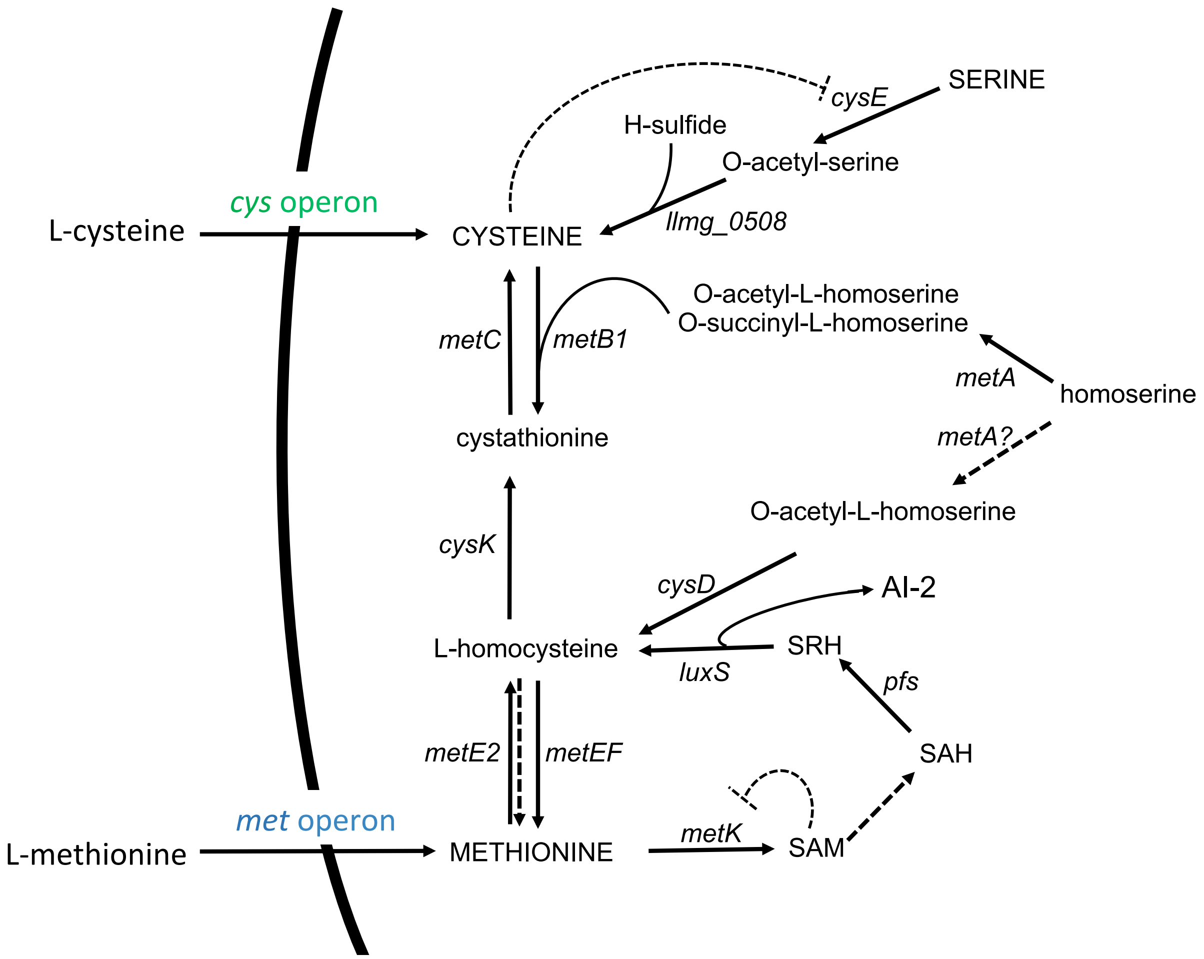

Supplementary Figure 4. **Transport and biosynthesis pathways of the sulfur-containing amino acids in *L. lactis* MG1363.** The cell via a putative transporter encoded in the *cys* operon can take up extracellular L-cysteine. In addition, L-cysteine can be intracellularly synthesized using methionine or serine as substrates. Firstly, serine is converted into O-acetyl-serine (OAS) by a serine O-acetyl transferase (encoded in the *cysE* gene), and OAS is subsequently converted into cysteine by activity of cysteine synthase (encoded in the gene *llmg_0508*). Secondly, MetC, using cysthathionine as a substrate, can synthesize L-cysteine. Cystathionine is obtained from methionine by activity of MetE2 and CysK enzymes, but also from homoserine by activity of the MetA and CysD enzymes. Although L-methionine can potentially be synthesized using L-homocysteine as a substrate, experimentally this bacterium is an auxotroph for L-methionine. The pool of homocysteine is derived from a recycling pathway via *metk, pfs* and *luxS*, or via interconversion of homoserine into L-homocysteine by *metA* and *cysD*. We speculate that either the amounts of L-methionine produced by these pathways are not

enough to let the bacteria proliferate or the biosynthetic pathways are impaired by gene mutations. In a review of the literature, Sperandio *et al*, 2010 described the sulfur amino acid metabolism in the *L. lactis* IL1403 strain, and showed that L-cysteine might enter by an interconversion pathway to L-methionine. This conversion is not possible in *L. lactis* MG1363 due to the lack of the enzyme YtjE that converts cystathionine into L-homocysteine. The Met transporter can obtain extracellular L-methionine. In this diagram, we highlighted the specific transporters for cysteine and methionine, other non-specific transporters for these amino acids (e.g. BcaP) are not shown.

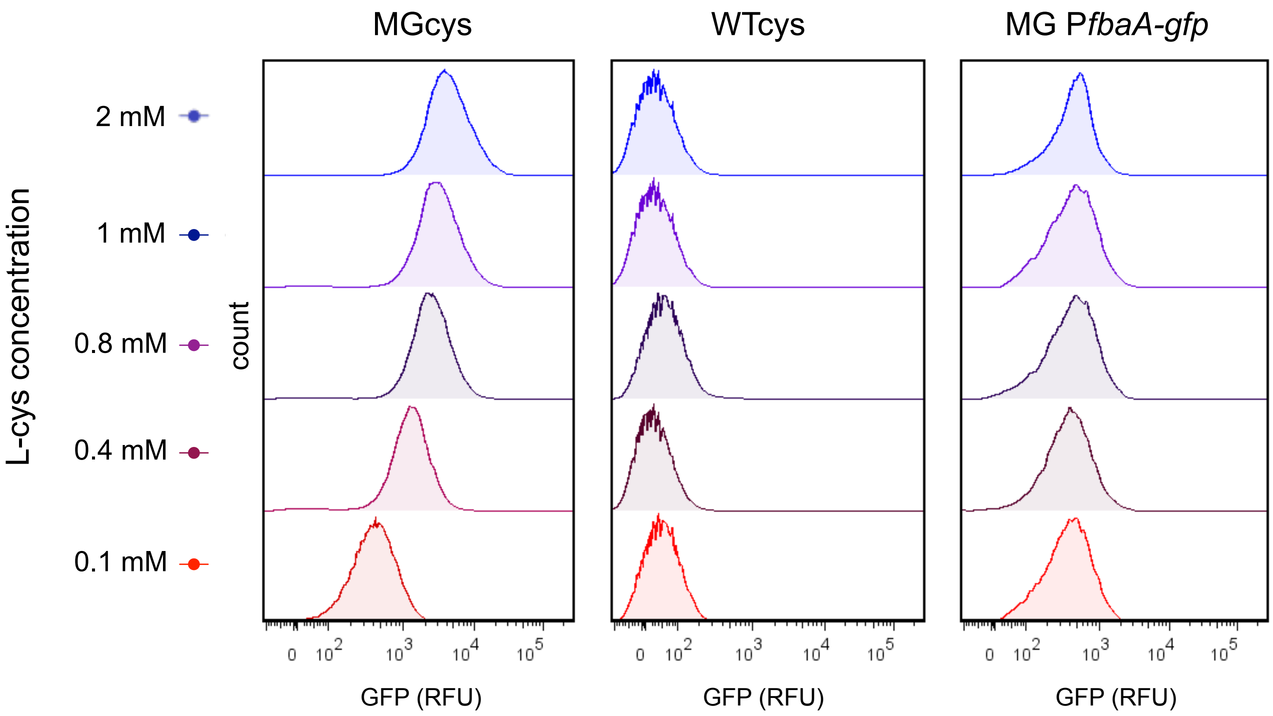

Supplementary Figure 5. Single-cell fluorescent measurement of the cysteine biosensor. Single-cell fluorescence measurements by flow cytometry, in the presence of increasing concentrations of cysteine (red to blue; 0.1-2 mM). Besides the strains carrying the P*cys-gfp* construct (MGcys and WTcys), a control *L. lactis* MG strain, with constitutive GFP expression (MG P*fbaA-gfp*), was used to discard the possibility that the growth conditions (cysteine concentration) or the cysteine effect on cell growth affect the GFP measurements. Fluorescence measurements were taken at the beginning of stationary growth phase. 10,000 ungated events for each sample are shown.

MGcys

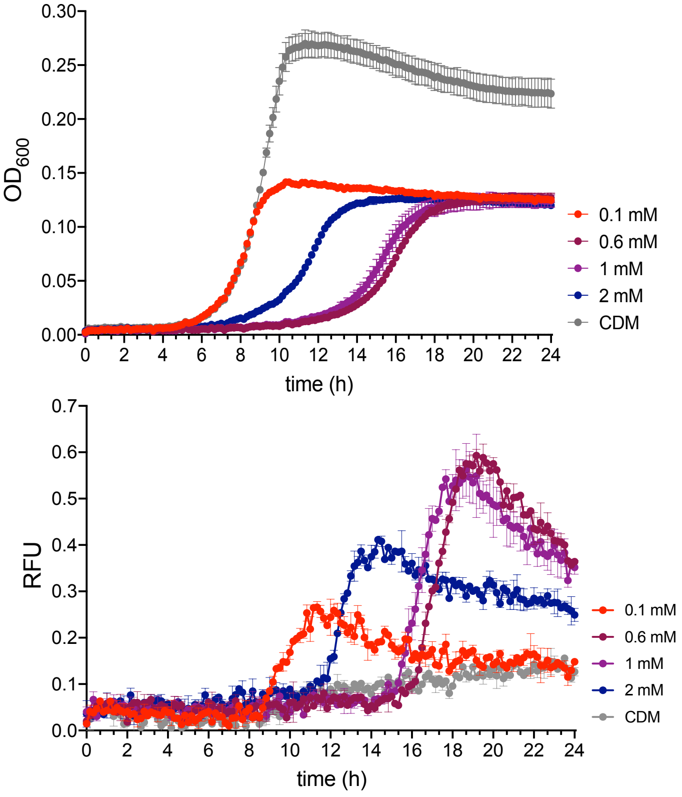

WTcys

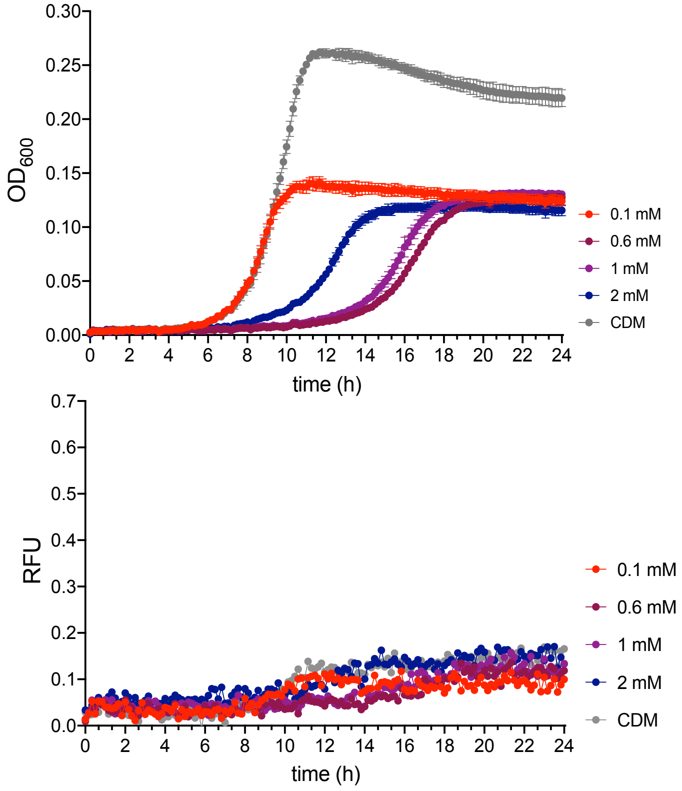

MG P*fbaA-gfp*

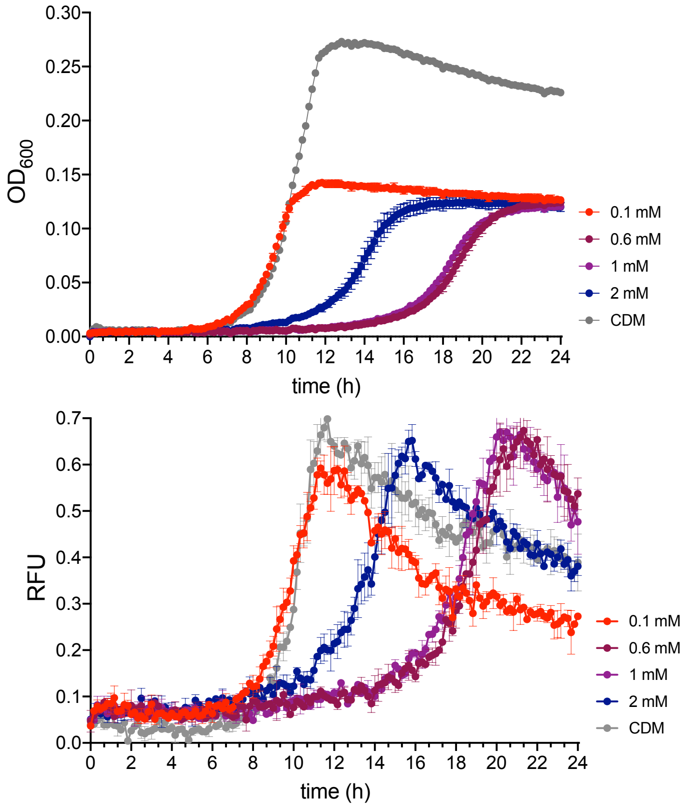

Supplementary Figure 6. Population-level GFP expression and growth measurements of the cysteine biosensors. The *L. lactis* strains MGcys, WTcys and MG P*fbaA-gfp* were grown in standard chemically defined medium (indicated as CDM, grey line), containing L-Met 0.27 mM and L-Cys 0.35 mM; and in CDM-cys, containing L-Met 0.07mM and supplemented with increasing cysteine concentrations (red to blue; 0.1-2 mM). Plots of the bacterial growth (top) and fluorescence measurements (bottom) obtained by plate reader assay are shown. Clear fluorescence peaks are observed. The maximum value of the fluorescence peak was corrected by the correspondent optical density (OD_{600}) value, yielding the relative fluorescent values (RFU/OD_{600}), as shown in the MGcys values (RFU/OD_{600}) in Figure 2 and Supplementary Figure 3 of this work. Points are means of 2 replicates for each growth curve. Data are presented as mean ± S.D. Error bars represent standard deviation (SD).

2 Supplementary Movies legends

Movie 1 – WTcys 0.07 mM L-Cys.

WTcys strain growing in CDM, containing L-Met 0.07 mM and L-Cys 0.07 mM. Time-lapse movie.

Movie 2 – WTcys 1 mM L-Cys.

WTcys strain growing in CDM, containing L-Met 0.07 mM and L-Cys 1 mM. Time-lapse movie.

Movie 3 – MGcys 0.07 mM L-Cys.

MGcys strain growing in CDM, containing L-Met 0.07 mM and L-Cys 0.07 mM. Time-lapse movie.

Movie 4 – MGcys 1 mM L-Cys.

MGcys strain growing in CDM, containing L-Met 0.07 mM and L-Cys 1 mM. Time-lapse movie.

Movie 5 – SK11&WTmet

WTmet (GFP+) and SK11 (GFP-) strains growing in CDMcasein, containing all amino acids, except L-Met. Time-lapse movie.

Movie 6 – WW4&WTmet

WTmet (GFP+) and WW4 (GFP-) strains growing in CDMcasein, containing all amino acids, except L-Met. Time-lapse movie.

Movie 7 – NCO176&WTmet

WTmet (GFP+) and NCDO176 (GFP-) strains growing in CDMcasein, containing all amino acids, except L-Met. Time-lapse movie.

Movie 8 – IPLA838&WTmet

WTmet (GFP+) and IPLA838 (GFP-) strains growing in CDMcasein, containing all amino acids, except L-Met. Time-lapse movie.

Movie 9 – WT PrtP+&WTmet

WTmet (GFP+) and WT PrtP+ (GFP-) strains growing in CDMcasein, containing all amino acids, except L-Met. Time-lapse movie.

Movie 10 – *codY* PrtP+&WTmet

WTmet (GFP+) and *codY* deletion mutant PrtP+ (GFP-) strains growing in CDMcasein, containing all amino acids, except L-Met. Time-lapse movie.

Movie 11 – *rel*PrtP+&WTmet

WTmet (GFP+) and *rel* deletion mutant PrtP+ (GFP-) strains growing in CDMcasein, containing all amino acids, except L-Met. Time-lapse movie.
